# A novel disulfidptosis-related lncRNA signature for predicting prognosis and potential targeted therapy in hepatocellular carcinoma

**DOI:** 10.1097/MD.0000000000036513

**Published:** 2024-01-26

**Authors:** Hui Zhang, Jiaojie Wang, Ming Yang

**Affiliations:** a Department of Breast Surgery, General Surgery Center, First Hospital of Jilin University, Changchun, China; b Department of Haematology, the First Hospital of Jilin University, Cancer Center, Changchun, Jilin, China.

**Keywords:** disulfidptosis, hepatocellular carcinoma, lncRNA, prognosis, signature, drug sensitivity

## Abstract

Disulfidptosis is a recently discovered mode of cell death with a significant role in cancer. Long non-coding RNAs (lncRNAs) have been implicated in numerous biological processes including oncogenesis, invasion, and metastasis. In this work, we developed an lncRNA signature associated with disulfidptosis for prediction of survival of hepatocellular carcinoma (HCC) patients. Detailed HCC expression profiles and clinical information were obtained from The Cancer Genome Atlas, and 599 differentially expressed disulfidptosis-related lncRNAs were identified through Pearson correlation analysis. Finally, by the least absolute shrinkage and selection operator method, we constructed an HCC prognostic model containing 7 disulfidptosis-related lncRNAs. We split patients into high- and low-risk groups based on the risk values generated by this model and showed that patients in the high-risk group had shorter overall survival times. In the training dataset, receiver operating characteristic curves for 1-, 3-, and 5-year survival were drawn according to the standard (0.788, 0.801, 0.803) and internal validation set (0.684, 0.595, 0.704) to assess the efficacy of the signature. Risk value was confirmed as an independent predictor and used to construct a nomogram in combination with several clinical factors. We further assessed the signature with respect to tumor immune landscape, gene set enrichment analysis, principal component analysis, tumor mutation burden, tumor immune dysfunction and exclusion, and drug sensitivity. High-risk patients had higher immune function scores, except for type II IFN response, whereas low-risk patients had significantly lower tumor immune dysfunction and rejection scores, indicating that they were more sensitive to immune checkpoint inhibitors. Drug sensitivity analysis showed that low-risk patients could benefit more from certain anti-tumor drugs, including sulafenib. In summary, we have constructed a novel signature that shows good performance in predicting survival of patients with HCC and may provide new insights for targeted tumor therapy.

## 1. Introduction

Hepatocellular carcinoma (HCC) accounts for more than 92% of liver neoplasms and represents a major disease burden worldwide,^[1,2]^ with liver cancer ranking third for incidence among common tumor types and approximately 746,000 patients dying from HCC every year.^[3,4]^ Despite remarkable advances in the treatment of liver neoplasms, including resection, transcatheter chemoembolization, transplantation, intra-arterial therapy, and other immunotherapy methods, the long-term survival rate of HCC patients remains very poor.^[5–7]^ Thus, there is a need for new predictive biomarkers and therapeutic targets to improve outcomes for HCC patients and optimize clinical management and treatment.

Disulfidptosis is a recently discovered mode of cell death that differs from common mechanisms such as apoptosis and ferroptosis. Disulfidptosis is closely associated with the actin cytoskeleton,^[8,9]^ a cellular structure with an important role in the maintenance of cell shape and survival. In tumor cells with high expression levels of SLC7A11, glucose deficiency induces a large accumulation of disulfide bonds, which leads to abnormal disulfide bond cross-linking and contraction of actin cytoskeletal proteins. This process ultimately leads to the breakdown of the actin network and cell death.^[10]^ Therefore, disulfidptosis could represent a source of potential targets for anti-tumor therapy and biomarkers for tumor diagnosis and prognosis. However, no study related to disulfidptosis and HCC has yet been reported.

Long non-coding RNAs (lncRNAs) are RNAs longer than 200 nucleotides that are not involved in encoding protein sequences.^[11,12]^ Accumulating evidence demonstrates that lncRNAs participate in regulation of proliferation, invasion, and metastasis of tumors via interactions with multiple biomolecules.^[13–15]^ For instance, lncRNA TLNC1 facilitates the proliferation and dissemination of hepatoma cells by inhibiting p53 signaling^[16]^; and KDM4A-AS1, a hypoxia-related lncRNA, has been identified as a potential target that could be used to promote the deterioration of HCC through the miR-411-5p/KPNA2/AKT axis.^[17]^ Therefore, it is worth exploring the potential value of disulfidptosis-related lncRNAs as prognostic markers and therapeutic targets.

In this study, we constructed a signature of 7 disulfidptosis-related lncRNAs to predict survival of HCC patients. Moreover, we performed gene set enrichment analysis (GSEA) and analyzed the relationships of the signature with the tumor immune infiltration landscape, tumor mutation burden (TMB), immunotherapy, and sensitivity to antineoplastic drugs in HCC.

## 2. Methods

### 2.1. Data acquisition

Ten disulfidptosis-related genes (*GYS1, NDUFS1, OXSM, LRPPRC, NDUFA11, NUBPL, NCKAP1, RPN1, SLC3A2*, and *SLC7A11*) were identified from the literature. Clinical information and other key data (mainly mRNA and lncRNA data) for the HCC samples used in this study were downloaded from The Cancer Genome Atlas (TCGA) (https://portal.gdc.cancer.gov/). The time needs to be within March 3, 2023. Any samples with incomplete clinical information or data were discarded. Expression matrices for mRNAs and lncRNAs were constructed using a Perl script. Pearson correlation analysis^[18]^ between the 10 disulfidptosis-related genes and lncRNAs from the TCGA database was performed to obtain disulfidptosis-related lncRNAs, using | R | ≥ 0.4 and *P* < .001 as thresholds. The “limma” package^[19]^ was used to identify differentially expressed lncRNAs between tumor tissue and adjacent normal tissues, using thresholds of |log_2_ fold change| > 1 and the false discovery rate < 0.05. Finally, we used cross-analysis to obtain differentially expressed disulfidptosis-related lncRNAs. Single-nucleotide variations in HCC were also downloaded from TCGA.

### 2.2. Construction and validation of signature

We randomly split the HCC patients into a training group and validation group with a ratio of 5:5 and used chi-squared tests to examine the discrepancies between samples in the 2 cohorts. We then used univariate analysis to extract the lncRNAs relevant to prognosis from the among differentially expressed disulfidptosis-related lncRNA, followed by least absolute shrinkage and selection operator (LASSO) and multivariable analyses to determine regression coefficients for the optimal prognostic model. Based on the prognostic signature, the following formula was used to calculate risk scores for the samples:


$$\begin{array}{l} Risk\;Score=\;\sum_{i=1}^{n}Expi\times Coei \end{array}$$

We classified patients into 2 groups (high-risk and low-risk) based on the above equation, with the median risk score as a cutoff, and used the “timeROC” package to plot receiver operating characteristic (ROC) curves to assess the accuracy of the signature. The “survminer” package was used to confirm the effectiveness of the signature among distinct clinical subgroups, and R package “scatterplot3d” for 3-dimensional principal component analysis.

### 2.3. Establishment of the nomogram

By univariate and multivariate analyses, we identified the risk score as an independent predictor using the “survival” package. Then, we used the “survival” and “rms” software packages to construct a nomogram for HCC survival prediction, incorporating the risk score together with clinical features such as age, gender, grade, and stage as parameters. Calibration curves for 1-, 3-, and 5-year survival were drawn to evaluate the consistency of predictions and overall survival (OS) using the “survcomp” package.

### 2.4. Functional enrichment analysis and GSEA

We subjected the differentially expressed genes between the high-risk group and low-risk group (identified using the “limma” package with criteria of |log_2_ fold change| > 1 and *P* < .05) to further functional enrichment analysis. Based on gene ontology and Kyoto Encyclopedia of Genes and Genomes analyses, we identified potential biological activities of these genes in HCC using the “clusterProfiler” package.^[20]^ The “enrichplot,” “ggpubr,” and “ggplot2” packages were also used to visualize the results of the analysis. In addition, GSEA was conducted to screen potential markers connected to the 7 risk-related lncRNAs using the “clusterProfiler” and “enrichplot” packages and the “c2.cp.kegg.Hs.symbols” file acquired from the MSigDB database.

### 2.5. Molecular mutations and tumor immune microenvironment

We used the “maftools” software package to analyze the gene mutations involved in HCC in the 2 risk groups and identified the most commonly altered genes. Differences in TMB between the 2 risk groups and OS status in the high- and low-TMB groups (L-TMB) were explored using the “limma” and “survival” packages. Furthermore, we used the “estimate” package to calculate ESTIMATE scores reflecting tumor purity for the 2 risk groups, and estimated the percentages of various types of immune cells with the CIBERSORT method. Finally, we explored immune cell populations and immune functions in the high- and low-risk groups using the “limma,” “GSVA,” “GSEABase,” and “ggpubr” R software packages.

### 2.6. Tumor immune dysfunction and rejection (TIDE) and drug sensitivity

Many studies have shown that lncRNAs have important roles in drug resistance and tumor progression in many types of cancer.^[21]^ For instance, Xia et al^[22]^showed that knockdown of lncRNA CCAT1 enhanced the sensitivity of enhanced HCC to oxaliplatin in vivo and in vitro. Given the availability of databases of drug response information, such as genomics of drug sensitivity in cancer, machine learning methods can be used to determine how different types of cancer cells respond to multiple cancer drugs, accelerating drug studies.^[23]^ Eftekhari et al used a high-dimensionality depletion approach based on matrix factorization, promoting the development of systemic pharmacology and precision medicine.^[24]^ Here, the TIDE website was used to compute TIDE scores for each HCC patients. Then, the “ggpub” and “limma” packages were used to further analyze and visualize the correlations between the signature and immunotherapy response. Drug data and all RNA sequencing read counts for HCC were acquired from the genomics of drug sensitivity in cancer database^[25]^; then, we used the “limma,” “oncoPredict,” “arallel,” and “ggplot2” packages to explore anti-tumor drug responses in patients with distinct risk scores. Half-maximal inhibitory concentration (IC_50_) values were used to indicate sensitivity to different anti-tumor drugs.

### 2.7. Statistical analyses

Our bioinformatics analysis relied on R software version 4.2.2. To translate gene names, identify lncRNAs, and generate expression matrices, we used Perl version 5.3.0. Pearson analysis was used to extract disulfidptosis-related lncRNAs, (coefficient ≥ 0.4 and *P* < .001) and chi-square test to explore discrepancies in clinical characteristics between patients in distinct risk groups. Univariate analysis, multivariable analysis, and LASSO regression were used for construction of the prognostic disulfidptosis-related lncRNA model. A *P* value <.05 was considered to indicate statistical significance.

## 3. Results

### 3.1. Data acquisition

**Figure 1.**
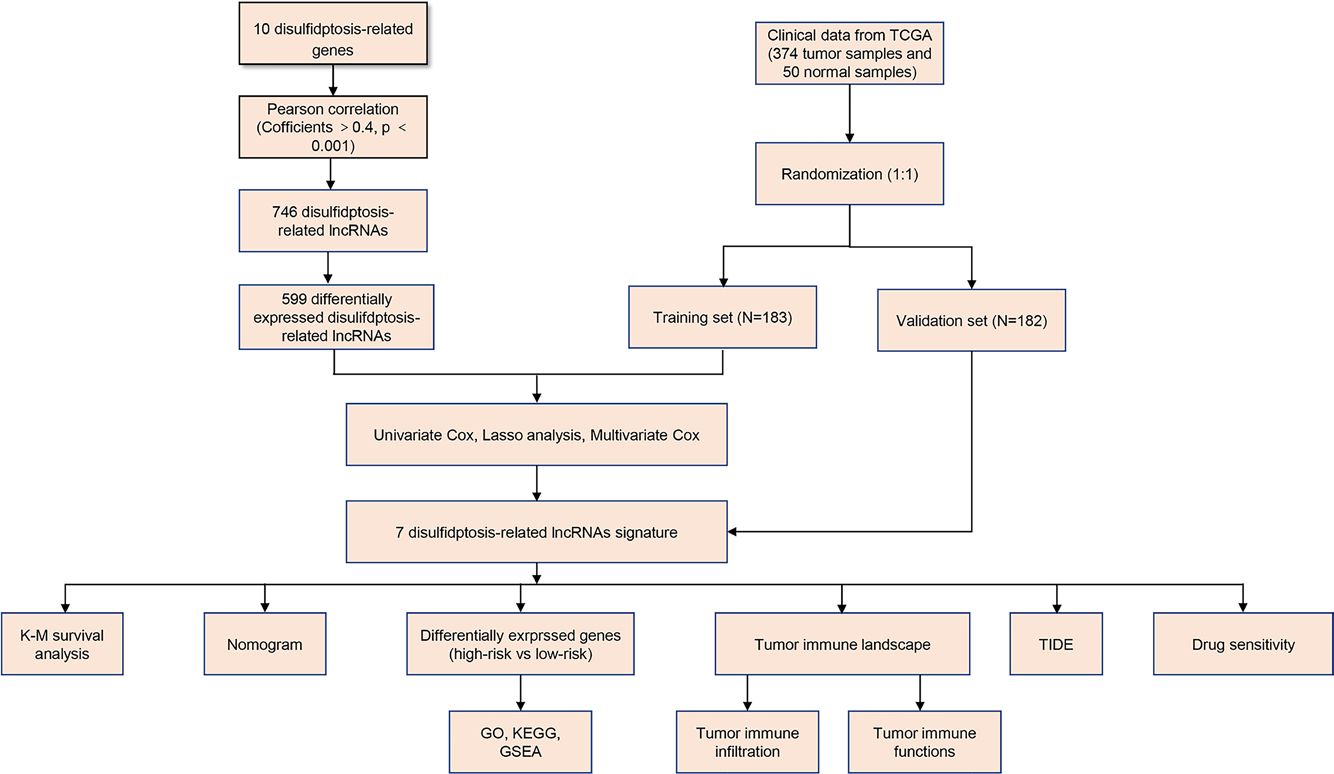
Schematic of the study process.

**Figure 2.**
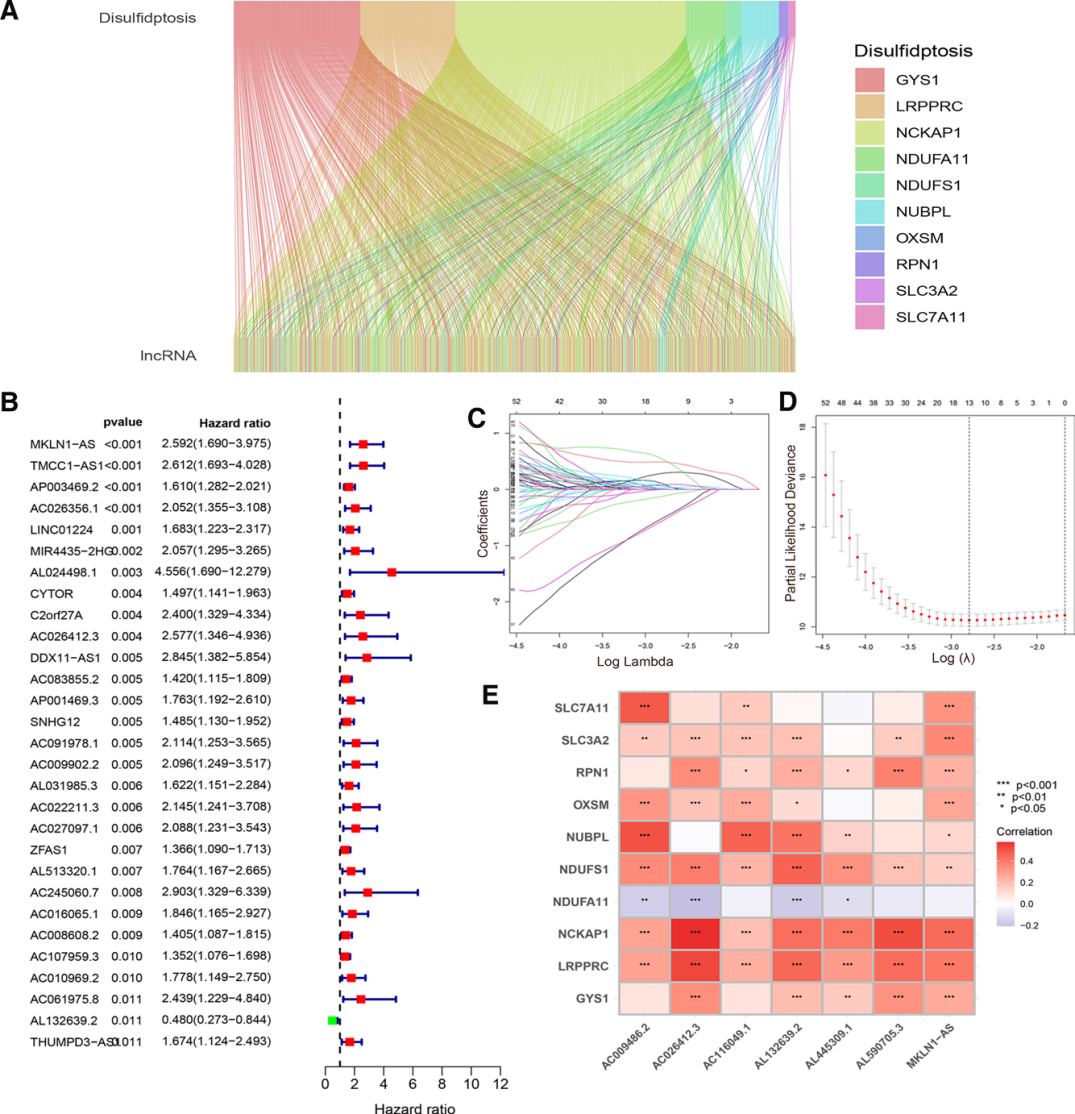
Construction of the signature. (A) Sankey diagram showing Pearson correlation analysis between ten disulfidptosis-related genes and lncRNAs. (B) Forest plot displaying the top 30 most statistically significant disulfidptosis-related lncRNAs. (C) LASSO coefficients of disulfidptosis-related lncRNAs. (D) Cross-validation of disulfidptosis-related lncRNAs in the LASSO regression. (E) Correlation analysis between the disulfidptosis-related genes and 7 lncRNAs of the signature. *P < .05, **P < .01, ***P < .001. lncRNA = long non-coding RNA, LASSO = least absolute shrinkage and selection operator.

Figure 1 shows a schematic of the study process. We collected 374 tumor samples and 50 corresponding normal adjacent tissues of HCC patients from TCGA through a clinical matrix. Based on randomization at a ratio of 5:5, we acquired a training set of 183 tumor samples and a validation set of 182 tumor samples (Supplemental Tables S1, http://links.lww.com/MD/L70, S2, http://links.lww.com/MD/L71). Chi-square tests demonstrated no difference in multiple clinical characteristics of patients between the training set and test set (Supplemental Table S3, http://links.lww.com/MD/L72). Perl was used to identify 19938 mRNAs and 16876 lncRNAs; Pearson correlation analysis generated 746 disulfidptosis-related lncRNAs (Fig. 2A, Supplemental Table S4, http://links.lww.com/MD/L73); and difference analysis resulted in 3747 differentially expressed lncRNAs. Then, 599 differentially expressed disulfidptosis-related lncRNAs (Supplemental Table S5, http://links.lww.com/MD/L74) were extracted for further analysis by taking the intersection of the differentially expressed and disulfidptosis-related lncRNAs.

### 3.2. Construction of the signature

First, 75 prognostic lncRNAs (Supplemental Table S6, http://links.lww.com/MD/L75) were identified through univariate analysis for differentially expressed disulfidptosis-related lncRNAs (*P* < .05). The forest plot in Figure 2B shows the top 30 most statistically significant lncRNAs. Then, 7 disulfidptosis-related lncRNAs were selected to develop a prognostic signature using the LASSO method in the training set (Fig. 2C and D). As shown in the correlation heatmap in Figure 2E, almost all risk-related lncRNAs had positive correlations with disulfidptosis-related genes, except for NDUFA11. A risk score was calculated for each patient using the following equation: risk score = (1.43885 × AC026412.3) + (0.44567 × AL590705.3) + (0.78672 × MKLN1-AS) + (−1.68929 × AL445309.1) + (−0.54330 × AL132639.2) + (0.42408 × AC116049.1) + (−1.81943 × AC009486.2). The median risk score was used as a cutoff to split patients with HCC into high- and low-risk groups.

**Figure 3.**
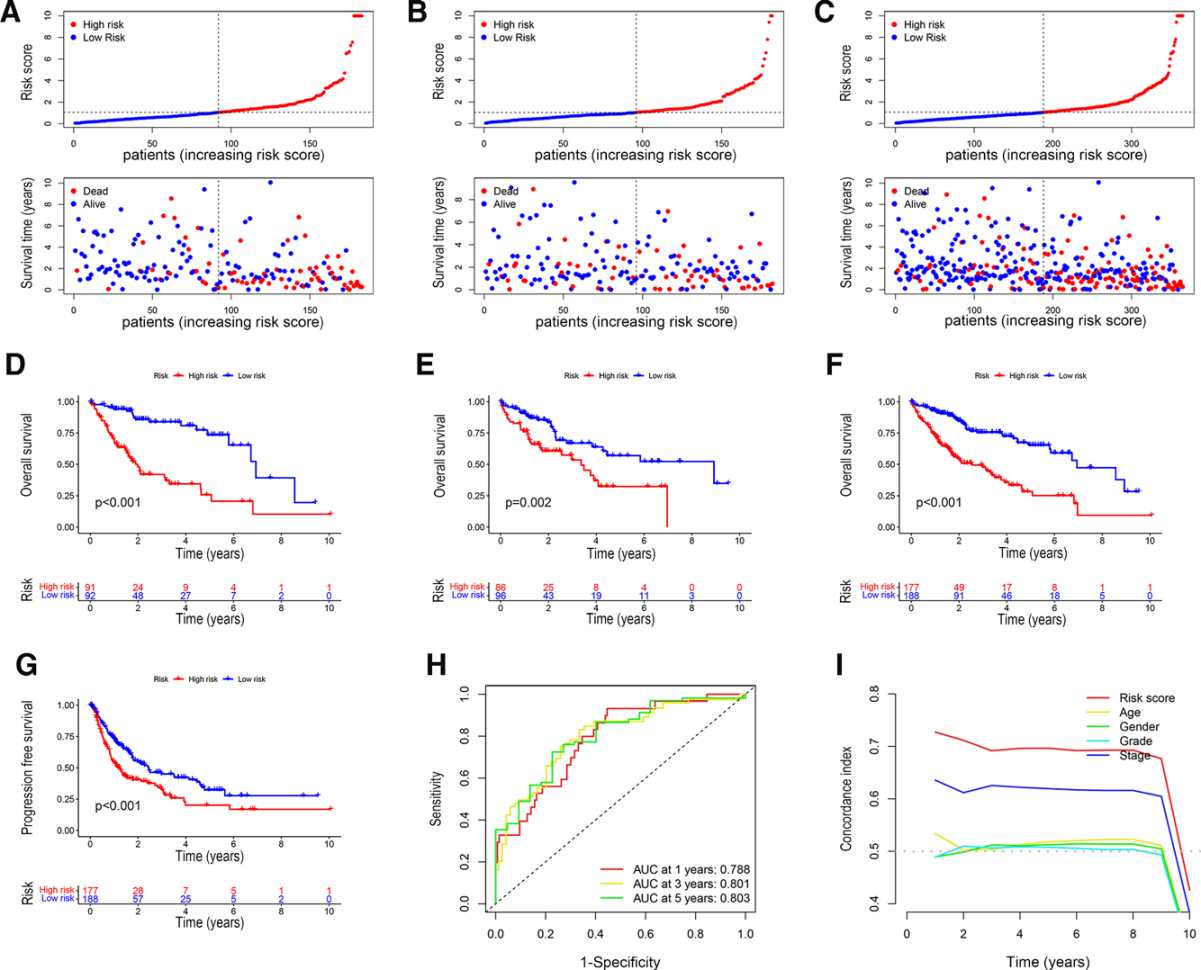
Validation of the signature. (A–C) Scatter plots of risk scores and overall survival (OS) status in the training cohort (A), validation cohort (B), and entire cohort (C). (D–F) K-M survival analysis of OS in the training set (D), validation set (E), and entire set (F). (G) K-M survival analysis of disease-free survival (DFS) in the entire set. (H) 1-, 3-, and 5-yr ROC curves for HCC patients in the training set. (I) Concordance index values for risk score and other clinicopathological characteristics. HCC = hepatocellular carcinoma, K-M = Kaplan–Meier, ROC = Receiver operating characteristic.

As shown in the scatter plots in Figure 3A–C, the OS time and survival probability for the training set, verification set, and the entire cohort were negatively correlated with risk scores. According to the Kaplan–Meier survival curves, patients in the high-risk group had relatively short OS and significantly shorter progression-free survival time compared with those in the low-risk group (Fig. 3D–G). The area under the ROC curve, representing the similarity of the predicted value to the actual value, was 0.788, 0.801, and 0.803 for 1-, 3-, and 5-year survival in the training set (Fig. 3H, Supplemental Fig. S1A and B, http://links.lww.com/MD/L68). The C-index plot for the signature further showed that the risk score had a higher C-index value than other clinical characteristics, indicating better predictive value (Fig. 3I). Detailed analysis of the ROC curves for the risk score and other common clinical features showed very high prediction ability for the risk score (Supplemental Fig. S1C, http://links.lww.com/MD/L68). Principal component analysis was performed to investigate the differences in expression profiles among the different risk groups and their clustering. The results indicated the reliability of the lncRNAs used to develop the signature (Supplemental Fig. S2, http://links.lww.com/MD/L69).

### 3.3. Clinical features confirm the signature

**Figure 4.**
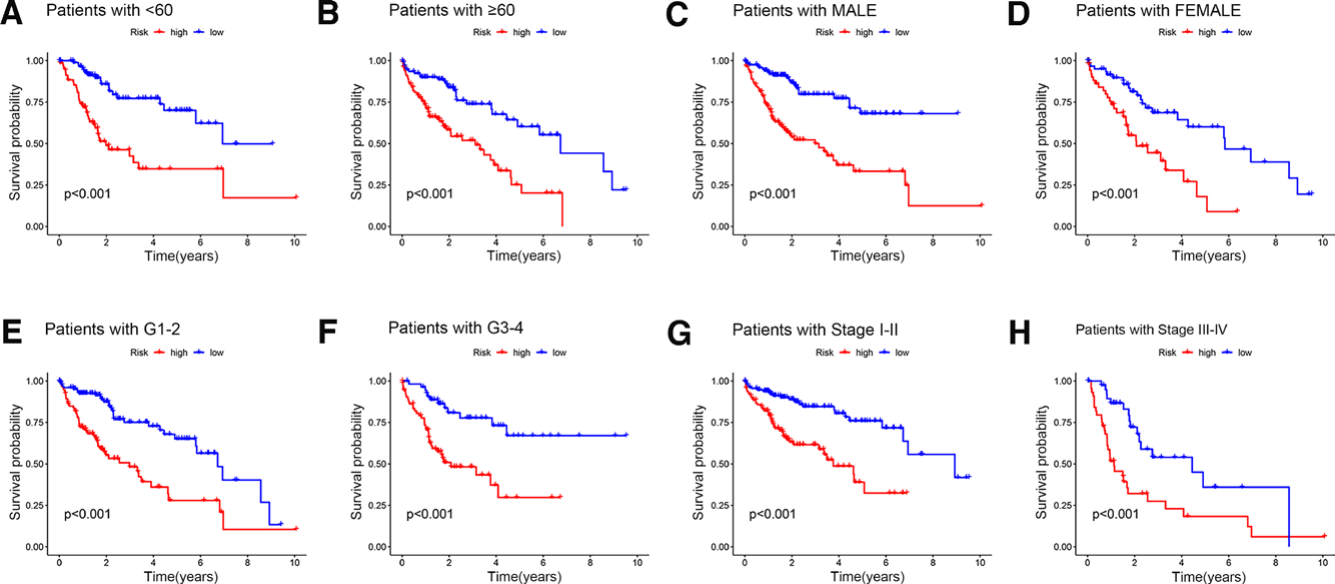
K-M survival analysis of overall survival in HCC patients in the high-risk group and low-risk group. (A and B) age; (C and D) gender; (E and F) grade; (G and H) tumor stage. HCC = hepatocellular carcinoma, K-M = Kaplan–Meier.

Based on the construction of a model of 7 lncRNAs closely related to disulfidptosis, we used survival analysis to analyze multiple clinical features of HCC patients (patients’ age, patients’ gender, actual grade, and specific stages). Compared with those in the low-risk group, patients in the high-risk group had significantly worse OS regardless of clinical factors (Fig. 4A–H). This analysis indicates that the prognostic model constructed this study could be applied to the analysis of various pathological features in the future.

### 3.4. Development of a nomogram

**Figure 5.**
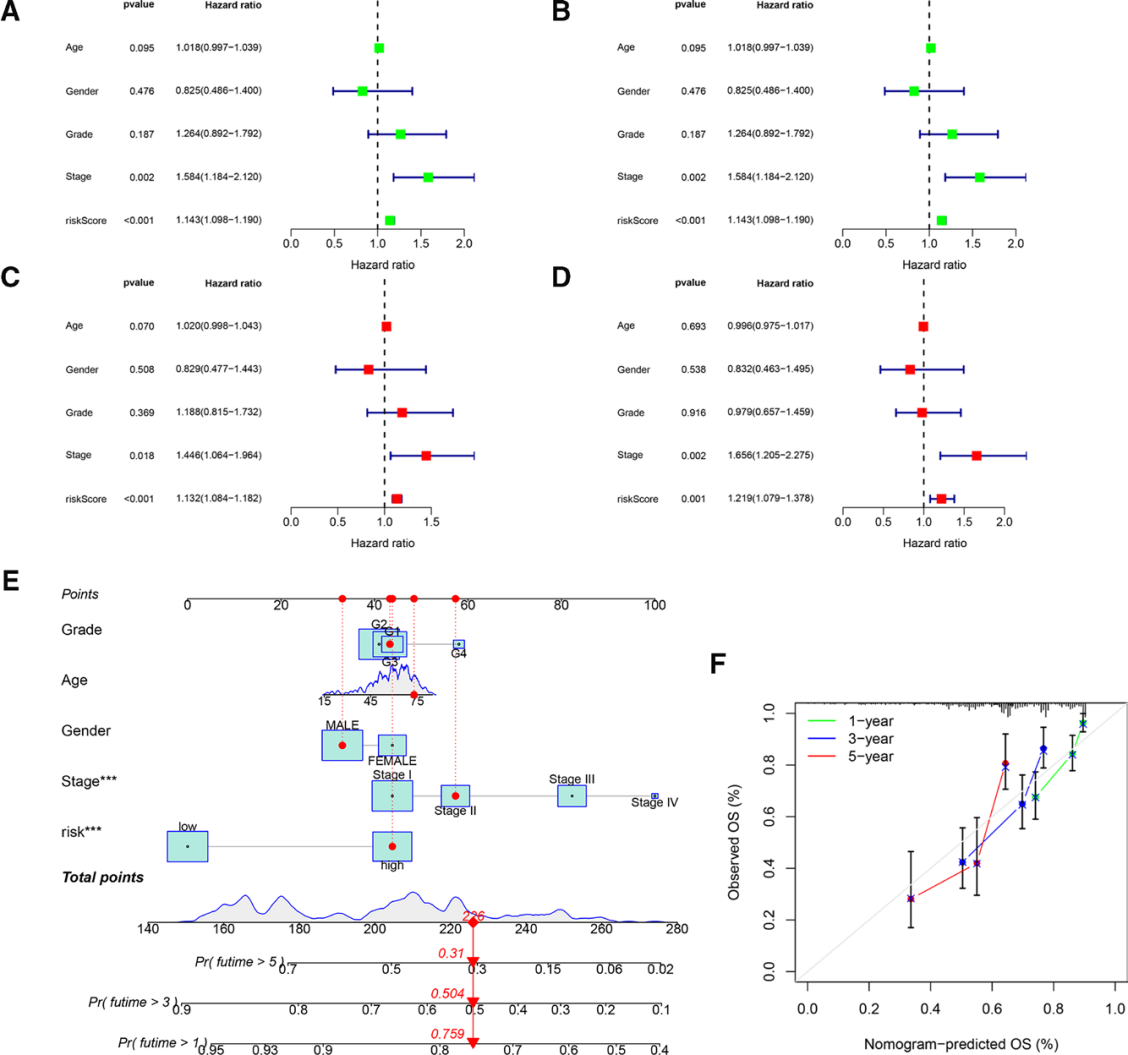
Construction of the nomogram. (A and C) Univariate and multivariate analyses confirm risk score as an independent prognostic factor in the training cohort. (B and D) Univariate and multivariate analyses confirm risk score as an independent prognostic factor in the internal validation cohort. (E) Construction of a nomogram combining the risk score with clinical parameters including grade, age, gender, and stage. (F) Calibration curves for 1, 3, and 5 yr demonstrating the accuracy of the nomogram.

Univariate and multivariate analyses were used to further confirm the predictive ability of the risk score and demonstrate that it is an independent predictor (Fig. 5A–D). Then, several clinicopathological parameters including age, gender, grade, and stage, together with the risk score, were used as parameters to develop a nomogram for prediction of the survival probability of the HCC population (Fig. 5E). Calibration curves reflected satisfactory accuracy of the nomogram (Fig. 5F), and our analysis indicated that the nomogram could perform well in predicting survival of HCC patients in clinical practice.

### 3.5. Functional annotation and GSEA

**Figure 6.**
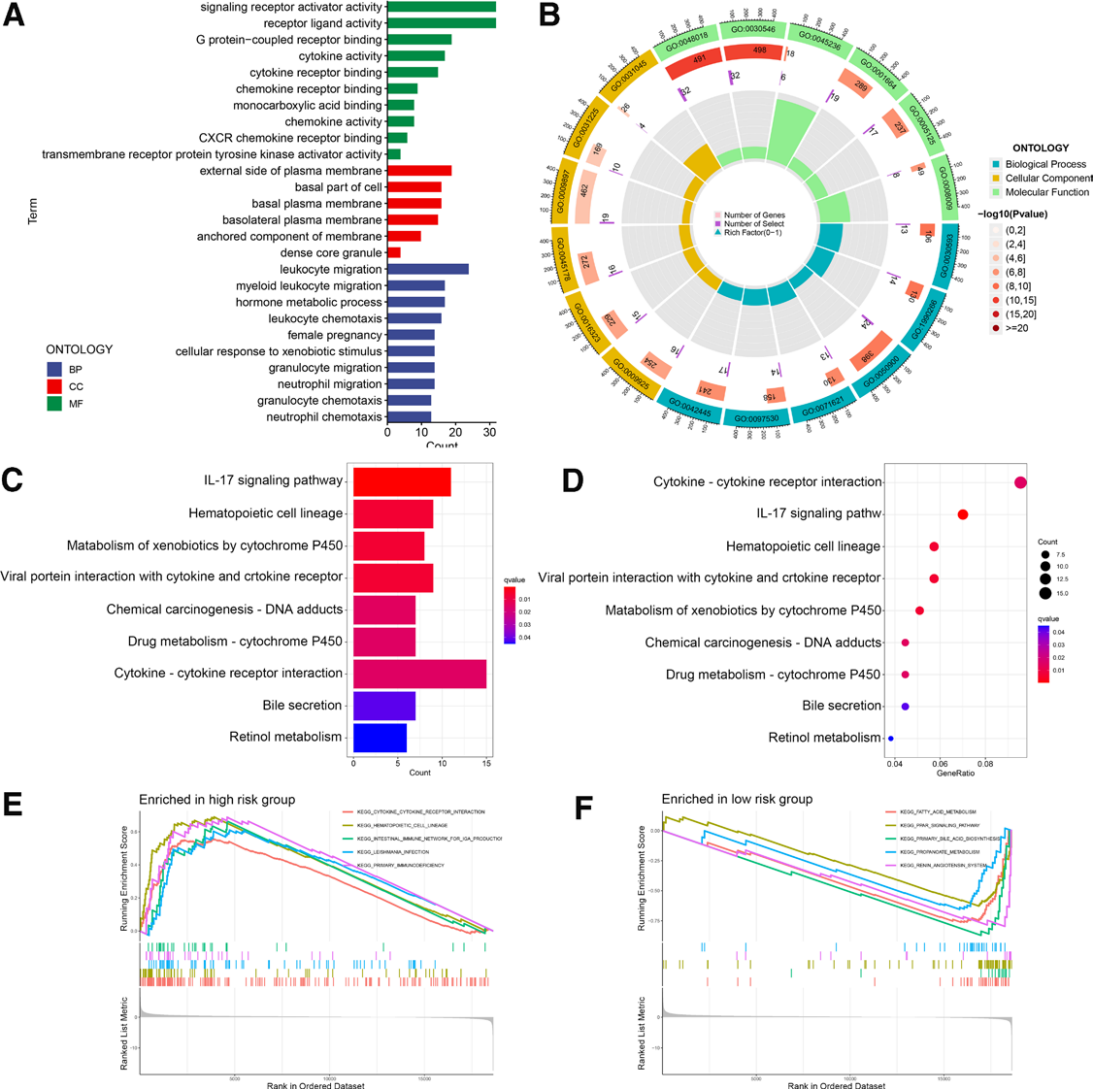
GO analysis and GSEA. (A and B) Bar plot and circle chart displaying GO enrichment terms of differentially expressed gene sets. (C and D) Bar plot and bubble diagram displaying significantly enriched pathways for differentially expressed gene sets. (E and F) GSEA demonstrating the top 5 enriched pathways in the high-risk and low-risk groups. GO = Gene ontology, GSEA = gene set enrichment analysis.

Through gene ontology analysis of the differentially expressed genes between the high-risk group and the low-risk group we identified the top 10 biological activities in the categories of cell components, biological processes (BP), and molecular functions (Fig. 6A and B). Among the molecular functions, signaling receptor activator activity and G protein-coupled receptor binding were remarkably enriched, whereas among the results relating to cell components were external side of plasma membrane and basal part of cell. Significantly enriched BPs included immunoregulatory-related BP such as leukocyte migration and myeloid leukocyte migration. In addition, Kyoto Encyclopedia of Genes and Genomes analysis of differentially expressed genes showed that they were abundant in cytokine − cytokine receptor interaction and the IL-17 signaling pathway. These pathways may contribute to tumorigenesis (Fig. 6C and D). Furthermore, the GSEA results suggested that hematopoietic cell lineage and primary immuno glucose were upregulated in the high-risk group (Fig. 6E), whereas metabolism-related signal transduction pathways such as primary bile acid biosynthesis and the renin angiotensin system were significantly enriched in the low-risk group (Fig. 6F). These results may contribute to our understanding of disulfidptosis and its impact on BPs.

### 3.6. Immune landscape and immune-related functional analyses

**Figure 7.**
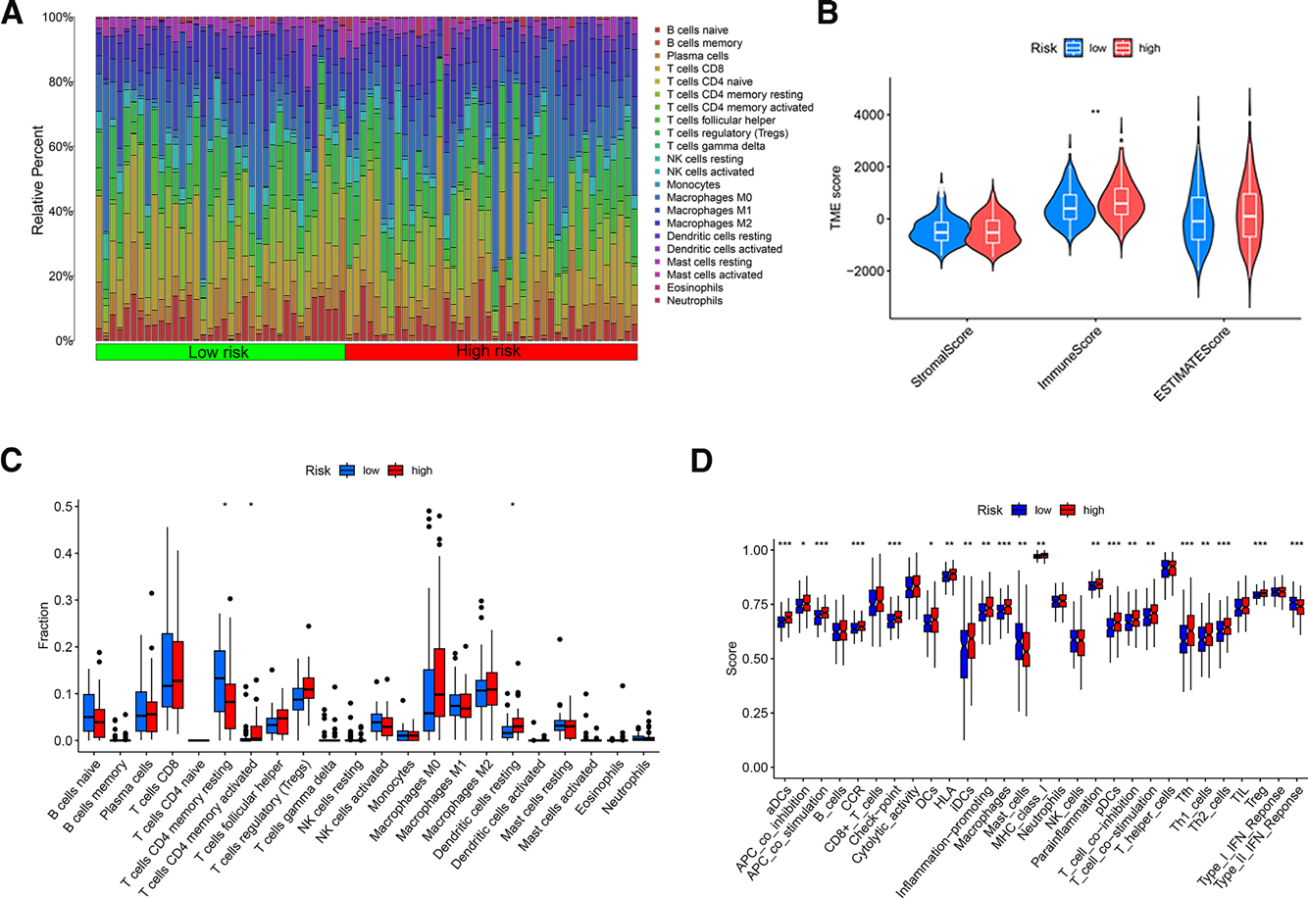
Tumor immune landscape and immune function analyses in HCC. (A) Percentages of tumor-infiltrating immune cells in HCC samples sorted by risk score. (B) TME component analysis. (C) Box plot showing differences in infiltrating immune cells between the high-risk and low-risk groups. (D) Box plot displaying differences in immune function between the high-risk and low-risk groups. *P < .05, **P < .01, ***P < .001. HCC = hepatocellular carcinoma, TME = tumor microenvironment.

We further investigated the connection between our signature and the tumor microenvironment (TME). Figure 7A shows the proportions of various tumor-infiltrating immune cell types in the HCC samples sorted according to risk score. CD8 and follicular helper T cells accounted for a relatively large percentage of immune cells. TME scores were calculated to explore the TME of HCC; the ESTIMATE score is equal to the stromal score plus the immune score. The immune scores of patients in the high-risk group were significantly higher compared with those in the low-risk group (Fig. 7B). However, as shown in the box chart in Figure 7C, there were significant discrepancies in immune cell proportions between distinct risk groups. We further analyzed the immune-related functions of the high- and low- risk groups by calculating an immune function score. With the exceptions of mast cells and type II IFN response, patients in the high-risk group had higher immune function scores (Fig. 7D). These results indicate that our model can accurately distinguish different tumor immune microenvironments, and can also accurately distinguish HCC patients with different immune-related functions.

### 3.7. Tumor mutation burden

**Figure 8.**
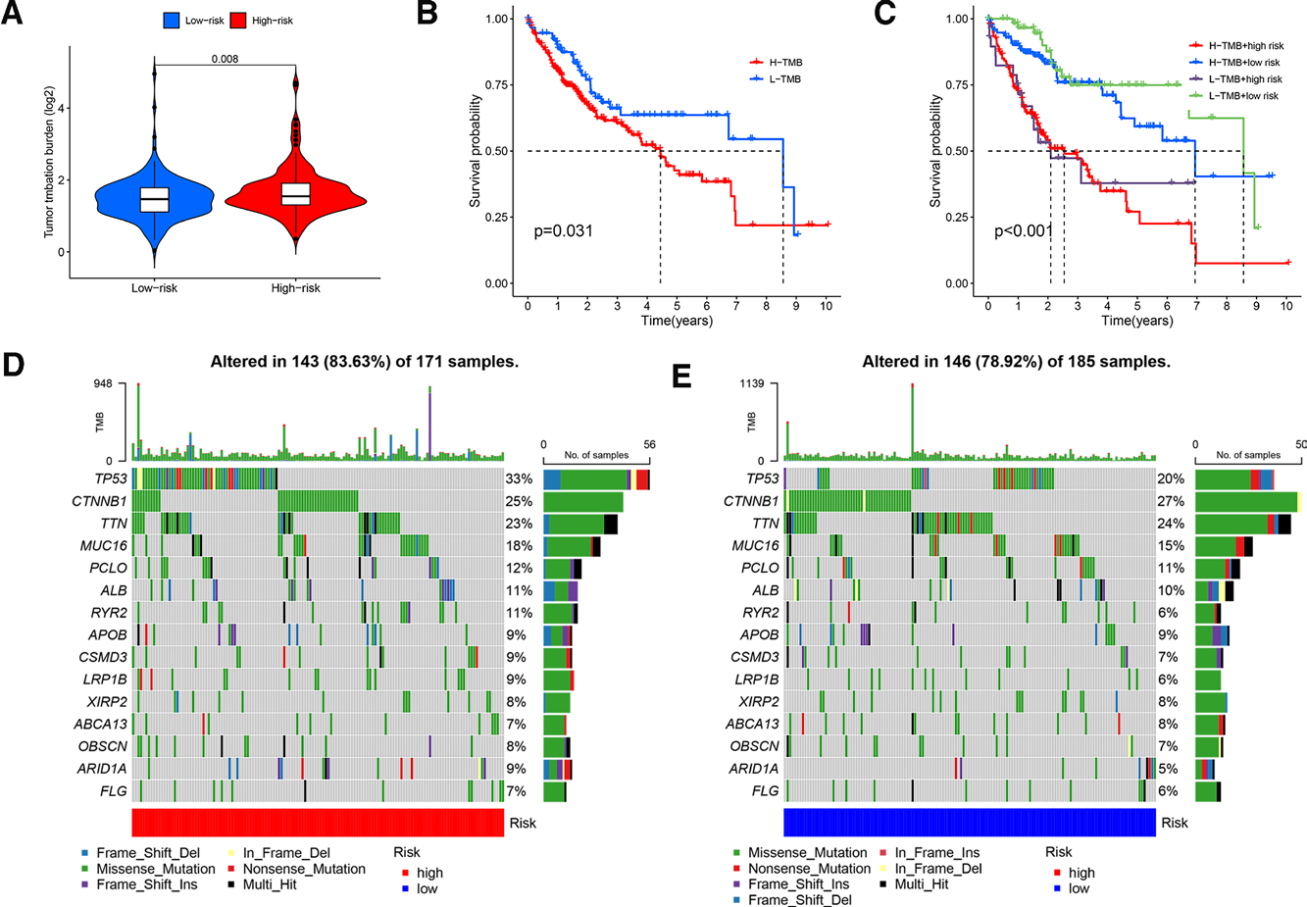
TMB analysis of samples in high-risk and low-risk cohorts. (A) Violin plot displaying TMB for different risk score groups based on the signature. (B) K-M survival analysis for H-TMB and L-TMB groups. (C) K-M survival analysis for H-TMB + high risk, H-TMB + low risk, L-TNM + high risk, and L-TNM + low risk groups. (D and E) Waterfall plot showing the top 15 most significantly altered genes in the high-risk (D) and low-risk (E) groups. K-M = Kaplan–Meier, TMB = tumor mutation burden.

TMB can be used to predict the effectiveness of immune checkpoint inhibitors against tumors. Figure 8A shows the differences in TMB between the high- and low-risk groups (*P* = .008). Based on mutation status, we divided patients into a high-TMB group and L-TMB. As shown in Figure 8B, patients in the high-TMB group group had worse OS prospects than those in the L-TMB group (*P* = .031). We combined the risk score with TMB to obtain new groups: H − TMB + high risk, L − TMB + high risk, H − TMB + low risk, and L − TMB + low risk. As shown in Figure 8C H-TMB patients had shorter OS than L-TMB patients, regardless of the risk score. The top 15 genes with the highest mutation rates were visualized using maftools plots (Fig. 8D and E). Genes *TP53* and *CTNNB1* had higher mutation frequencies in the low-risk group, whereas the mutation rates of *TP53, MUC16*, and *PCLO* were higher in the high-risk group.

### 3.8. TIDE and drug sensitivity analysis

**Figure 9.**
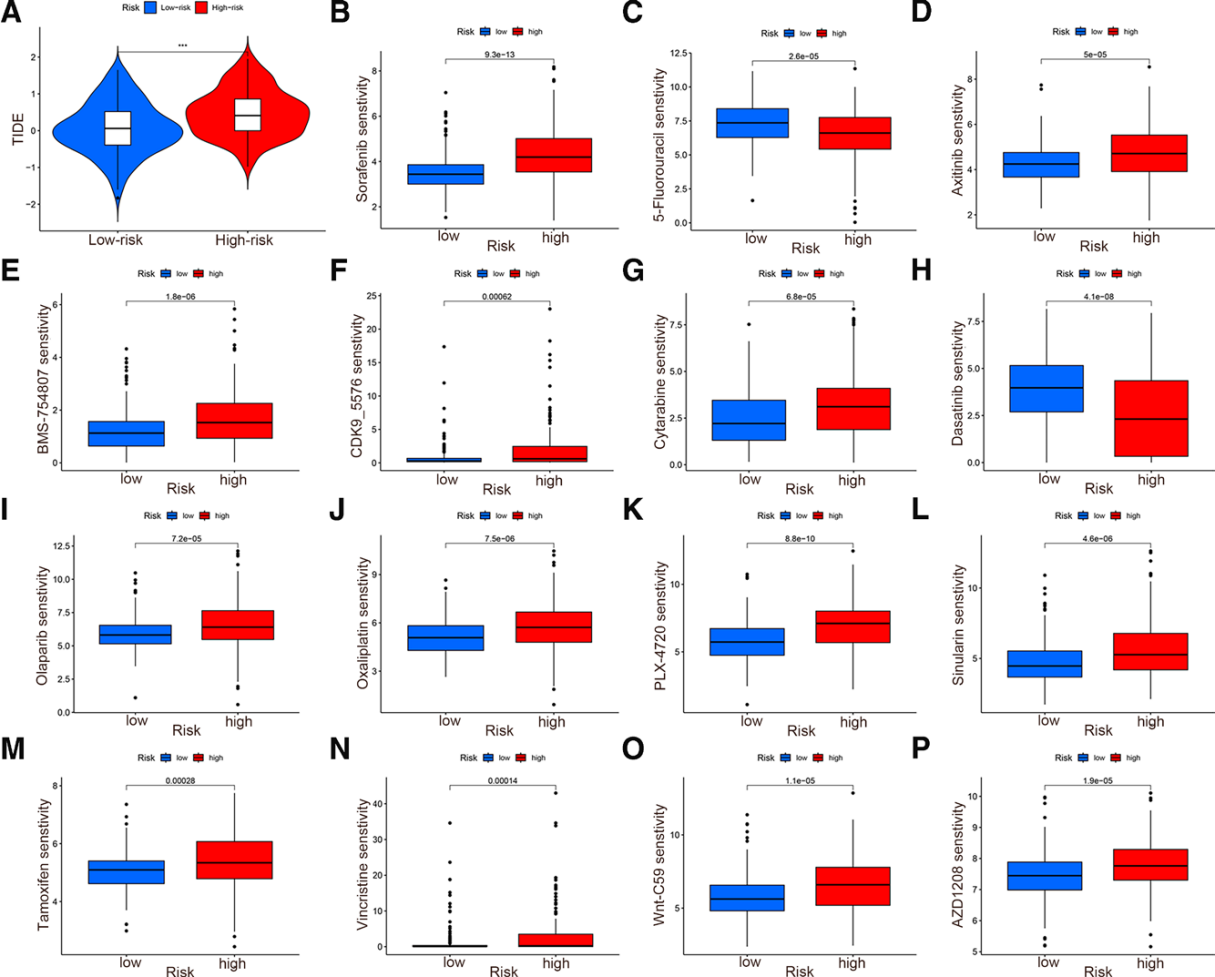
TIDE and drug sensitivity analyses based on the signature. (A) Violin plot indicating that patients in the high-risk group are more likely to escape immunotherapy. (B–P) Drug sensitivity analyses for sorafenib (B), 5-fluorouracil (C), axitinib (D), BMS-754807 (E), CDK9_5576 (F), cytarabine (G), dasatinib (H), olaparib (I), oxaliplatin (J), PLX-4720 (K), sinularin (L), tamoxifen (M), vincristine (N), Wnt-C59 (O), and AZD1208 (P). TIDE = tumor immune dysfunction and rejection.

The TIDE algorithm is used to assess the possibility of tumor immune escape. TIDE scores reflect sensitivity to immune checkpoint inhibitors and can thus be used to predict the immunotherapy response of tumors. As shown in the violin plot in Figure 9A, HCC patients in the low-risk group had lower TIDE scores than those in the high-risk group, suggesting that the low-risk patients would be more sensitive to immunotherapy. Furthermore, we conducted drug sensitivity analysis at the genomic level and identified several anti-tumor drugs to which HCC showed a distinct therapeutic response in the high- and low-risk groups (Fig. 9B–P). Lower IC_50_ values indicate stronger sensitivity to the drug, and greater specificity.

## 4. Discussion

Numerous studies have demonstrated the close association of lncRNAs with proliferation, infiltration, and metastasis of multiple tumor types.^[13–15]^ Moreover, lncRNAs with different phenotypes are increasingly being used in the construction of prognostic signatures, displaying satisfactory predictive performance.^[26,27]^ Various anti-cancer treatments kill malignant tumor cells through apoptosis. However, many carcinomas seek escape from treatment-induced apoptosis, resulting in treatment resistance and recurrence. Recently, disulfidptosis, a mode of cell death that differs from ferroptosis, cuproptosis, necroptosis, and autophagy, was discovered.^[8,10]^ Disulfidptosis occurs under glucose starvation conditions, when excessive accumulation of cystine leads to disulfide stress in cancer cells with overexpression of SLC7A11, which further causes rapid cell death. Glucose uptake inhibitors can induce disulfidptosis of tumor cells with overexpression of SLC7A11, thus delaying cell proliferation without significant side effects. This breakthrough discovery could result in new cancer treatment methods and provide novel prognostic biomarkers for carcinomas. However, there has not yet been any study on disulfidptosis and HCC.

In the present study, a signature of 7 disulfidptosis-related lncRNAs identified in HCC patients was constructed using the LASSO method, univariate analysis, and multivariate analysis. HCC patients were split into high- and low-risk groups based on the median value of the risk score. Patients in the high-risk group were found to have worse prognostic prospects. The model was confirmed to have good reliability compared with various clinicopathological features, including gender, age, stage, and grade, and ROC curves indicated that it has good ability to predict the prognosis of HCC patients. Indeed, the risk score was shown to be a better predictor than other clinicopathological parameters and confirmed as an independent predictor. We further combined the risk score clinicopathological characteristics to develop a nomogram, and calibration curves displayed strong consistency between nomogram predictions and the actual probability.

The prognostic signature developed in our study consists of 7 disulfidptosis-related lncRNAs, AC026412.3, AL590705.3, MKLN1-AS, AL445309.1, AL132639.2, AC116049.1, and AC009486.2. Recent studies have shown that MKLN1-AS is overexpressed in HCC, and that its upregulation can serve as a marker of adverse prognosis.^[28,29]^ MKLN1-AS can enhance the expression of hepatoma-derived growth factor by functioning as a competitive endogenous RNA for miR-654-3p, which promotes deterioration of HCC. Thus, the MKLN1-AS/hepatoma-derived growth factor pathway could represent a novel potential prognostic predictor and therapeutic target. Similarly, MKLN1-AS is associated with malignant characteristics of HCC cells via the MKLN1-AS/ETS1 regulatory axis,^[30]^ and Guo et al showed that it functions as an upstream factor of Yes-associated transcriptional regulator 1, thereby aggravating the proliferation, migration, and invasion of HCC.^[31]^ According to another study, the SOX9/MKLN1-AS axis promotes HCC cell proliferation and epithelial–mesenchymal transition through transcriptionally regulating MKLN1-AS.^[32]^ In addition, Zhang et al identified MKLN1-AS as an important component of HCC prognostic models.^[33]^ All these findings, which are in line with the results of our bioinformatics analyses, indicate that MKLN1-AS could serve as a potential marker for diagnosis and prognosis, as well as a therapeutic target in HCC patients. The Wei et.al find that the inhibition of lncRNA AL590705.3 expression decreased the proliferative capacity of HCC cells but enhanced FDX1 expression.^[34]^ AL590705.3 may affect HCC progression by participating in the occurrence of cuproptosis. AL590705.3 has been confirmed to serve as a prognostic predictor in multiple tumor types, including gastric cancer and HCC.^[35,36]^ Previous bioinformatics analysis have also demonstrated the potential of AL590705.3 as a prognostic biomarker or therapeutic target.^[37,38]^ Previous studies^[39–42]^ have used AC026412.3 in the development of multiple prognostic models of HCC with satisfactory performance. AC026412.3 is differentially expressed between HCC and normal tissues and has been linked to poor prognosis of HCC patients.^[43,44]^ There has not yet been any research on AL445309.1, AL132639.2, AC116049.1, and AC009486.2; however, our study might assist in understanding the potential functions of these 4 lncRNAs.

With the gradual development of immunotherapy for HCC,^[45]^ further exploration of the immune infiltration landscape is valuable. Our results showed obvious differences in the expression levels of CD4 memory resting and CD4 memory activated T cells in different risk groups. A phase I clinical study has suggested that activated and expanded autologous tumor-infiltrating immune cells may achieve good therapeutic effects in HCC patients.^[46]^ Here, high-risk patients had higher ImmuneScore values according to the ESTIMATE algorithm. Immune function analysis also resulted in higher scores for most immune functions, except for mast cells and type II IFN response. The discrepancies in abundance of infiltrating immune cells and immune function may provide some information for future exploration of immunotherapy for HCC. Moreover, patients in the high-risk group had higher TIDE scores; this finding may assist clinician in identifying patients who are more suitable for receiving immune checkpoint inhibitor therapy.

Moreover, drug sensitivity analysis was implemented to promote the discovery of new therapeutic biomarkers for HCC treatment. For example, HCC patients in the low-risk group were found to be more sensitive to sorafenib. Sorafenib is the first approved targeted drug for advanced HCC and shows definite therapeutic effects. This drug sensitivity information may provide some guidance for clinical practice.

Our work had some limitations. First, this analysis lacked in vitro experiments to investigate the specific molecular mechanisms of the lncRNAs identified. Second, due to the absence of external database data, the study also lacks external validation; multi-center, large-scale, real-world sample data are required to further confirm the predictive significance of disulfidptosis-related lncRNAs in HCC.

## Acknowledgments

We were grateful for the initial data provided on the TCGA website.

## Author contributions

**Conceptualization:** Hui Zhang, Jiao Jie Wang.

**Data curation:** Hui Zhang, Jiao Jie Wang.

**Investigation:** Hui Zhang.

**Software:** Hui Zhang.

**Supervision:** Jiao Jie Wang.

**Validation:** Jiao Jie Wang.

**Writing – original draft:** Jiao Jie Wang.

**Writing – review & editing:** Ming Yang.

## Supplementary Material
















