# Supplementary material for: A novel disulfidptosis-related lncRNA signature for predicting prognosis and potential targeted therapy in hepatocellular carcinoma
Source: Medicine (Baltimore). 2024 Jan 26;103(4):e36513. doi: 10.1097/MD.0000000000036513 (PMC10817158; doi:10.1097/MD.0000000000036513)
Supplement: Supplementary file 3 [file medi-103-e36513-s003.docx]

**Table 3.** Clinical characteristics of patients in training and validation sets

| **Covariates** |  | **Validation cohort (N=182)** | **Training cohort (N=183)** | **Pvalue** |
| --- | --- | --- | --- | --- |
| Age | <=60 | 84(46.15%) | 89(48.63%) | 0.7117 |
|  | >60 | 98(53.85%) | 94(51.37%) |  |
| Gender | FEMALE | 55(30.22%) | 64(34.97%) | 0.3915 |
|  | MALE | 127(69.78%) | 119(65.03%) |  |
| Grade | G1 | 28(15.38%) | 27(14.75%) | 0.9325 |
|  | G2 | 89(48.9%) | 86(46.99%) |  |
|  | G3 | 58(31.87%) | 60(32.79%) |  |
|  | G4 | 5(2.75%) | 7(3.83%) |  |
|  | unknow | 2(1.1%) | 3(1.64%) |  |
| Stage | Stage I | 86(47.25%) | 84(45.9%) | 0.7917 |
|  | Stage II | 45(24.73%) | 39(21.31%) |  |
|  | Stage III | 38(20.88%) | 45(24.59%) |  |
|  | Stage IV | 2(1.1%) | 2(1.09%) |  |
|  | unknow | 11(6.04%) | 13(7.1%) |  |
| T | T1 | 91(50%) | 89(48.63%) | 0.1657 |
|  | T2 | 46(25.27%) | 45(24.59%) |  |
|  | T3 | 34(18.68%) | 44(24.04%) |  |
|  | T4 | 10(5.49%) | 3(1.64%) |  |
|  | unknow | 1(0.55%) | 2(1.09%) |  |
| M | M0 | 134(73.63%) | 129(70.49%) | 1 |
|  | M1 | 2(1.1%) | 1(0.55%) |  |
|  | unknow | 46(25.27%) | 53(28.96%) |  |
| N | N0 | 130(71.43%) | 118(64.48%) | 1 |
|  | N1 | 2(1.1%) | 2(1.09%) |  |
|  | unknow | 50(27.47%) | 63(34.43%) |  |
