# Supplementary figures and images for: A novel disulfidptosis-related lncRNA signature for predicting prognosis and potential targeted therapy in hepatocellular carcinoma
Source: Medicine (Baltimore). 2024 Jan 26;103(4):e36513. doi: 10.1097/MD.0000000000036513 (PMC10817158; doi:10.1097/MD.0000000000036513)

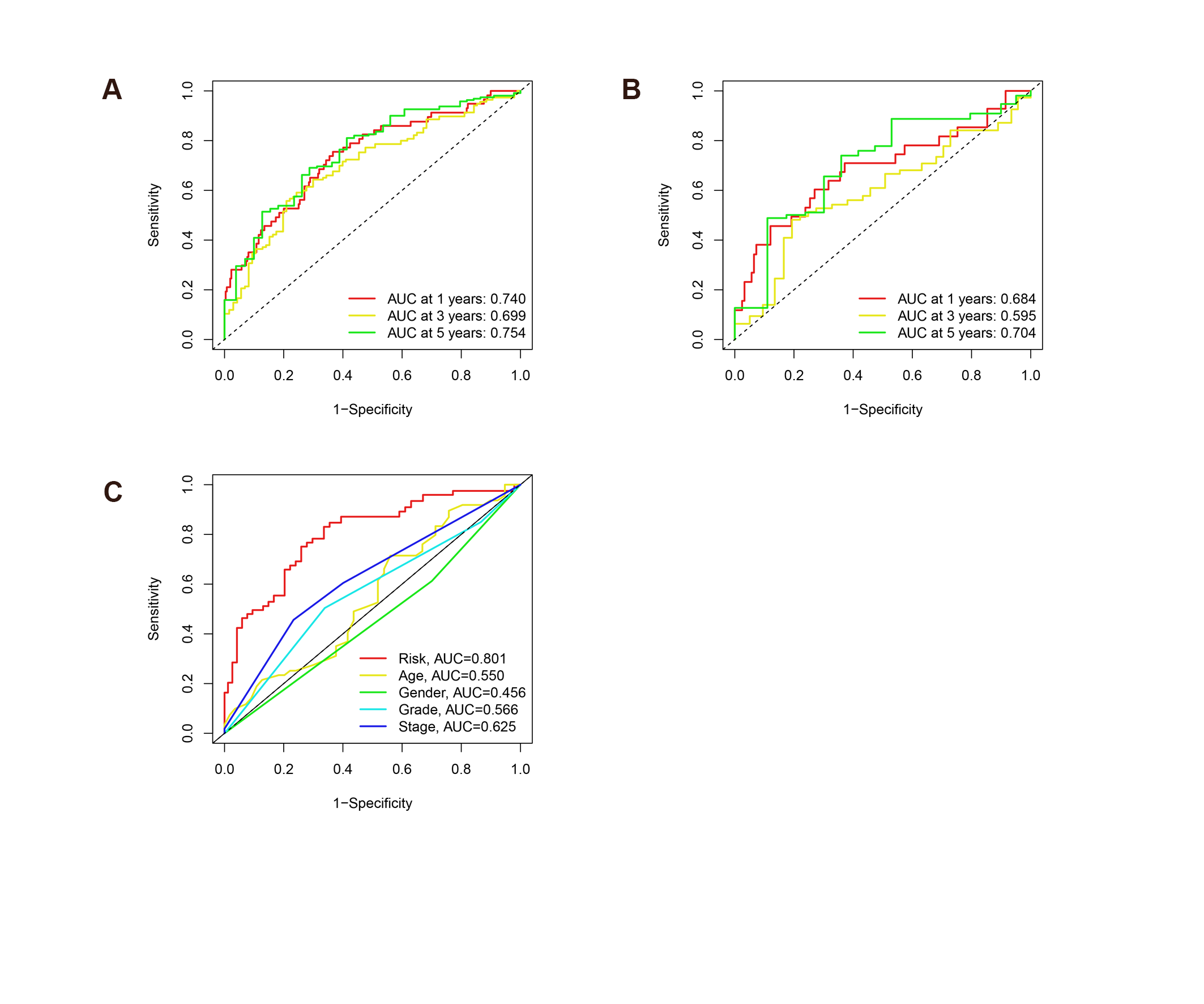

Supplement: Supplementary file 7 [file medi-103-e36513-s007.tif]

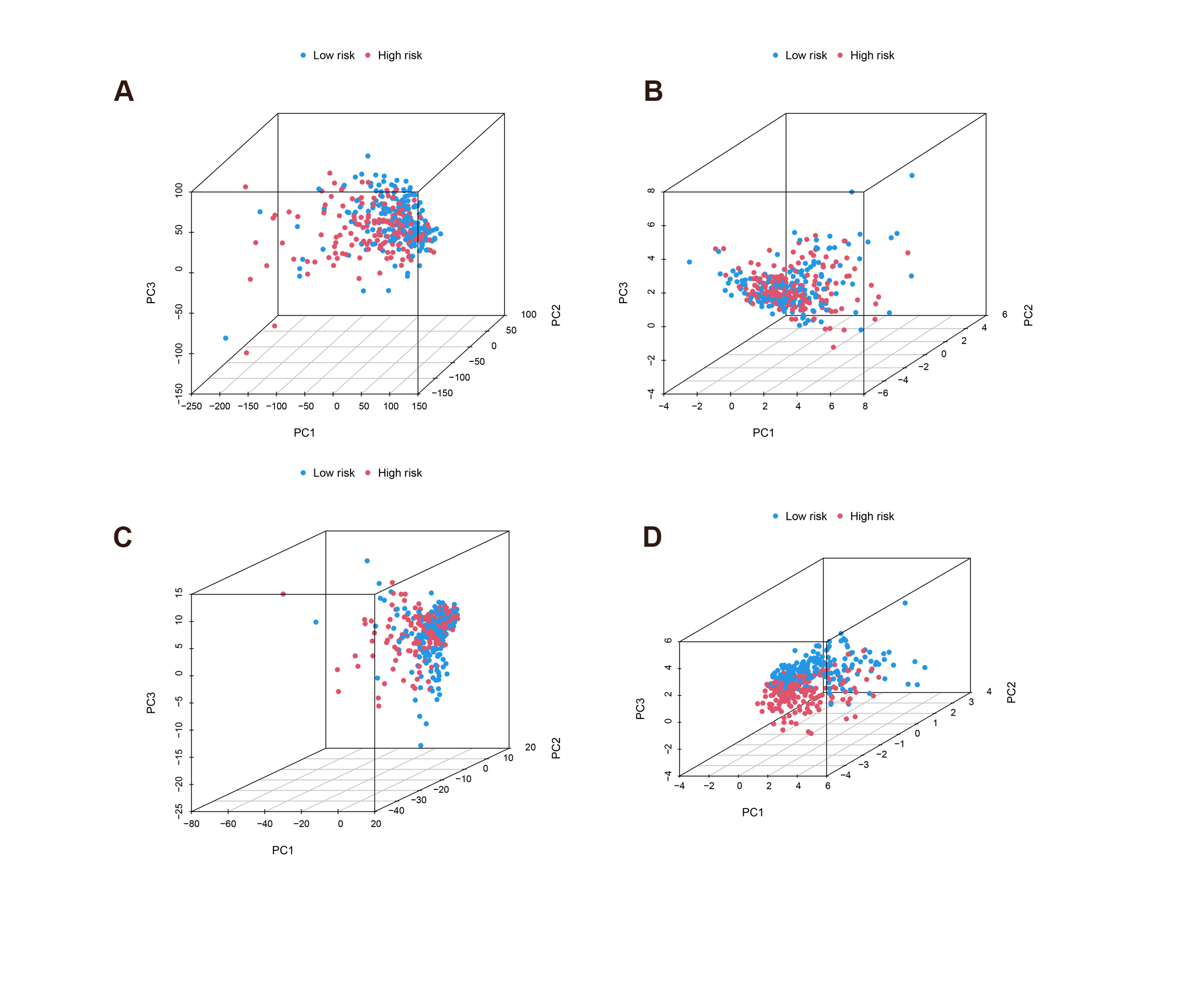

Supplement: Supplementary file 8 [file medi-103-e36513-s008.tif]
